# Suppressing Tau Aggregation and Toxicity by an Anti-Aggregant Tau Fragment

**DOI:** 10.1007/s12035-018-1326-z

**Published:** 2018-09-08

**Authors:** Ghulam Jeelani Pir, Bikash Choudhary, Senthilvelrajan Kaniyappan, Ram Reddy Chandupatla, Eckhard Mandelkow, Eva-Maria Mandelkow, Yipeng Wang

**Affiliations:** 1 German Center for Neurodegenerative Diseases (DZNE), Sigmund-Freud-Str. 27, 53127 Bonn, Germany; 2 Max-Planck-Institute for Metabolism Research, Hamburg Outstation, c/o DESY, Notkestrasse 85, 22607 Hamburg, Germany; 3 CAESAR Research Center, Ludwig-Erhard-Allee 2, 53175 Bonn, Germany

**Keywords:** Aggregation, Alzheimer disease, β-breaker peptides, Cell model, Microtubules, Tau, Transgenic *C.elegans*, Life-span

## Abstract

Tau aggregation is a hallmark of a group of neurodegenerative diseases termed Tauopathies. Reduction of aggregation-prone Tau has emerged as a promising therapeutic approach. Here, we show that an anti-aggregant Tau fragment (F3^ΔKPP^, residues 258–360) harboring the ΔK280 mutation and two proline substitutions (I^277^P & I^308^P) in the repeat domain can inhibit aggregation of Tau constructs in vitro, in cultured cells and in vivo in a *Caenorhabditis elegans* model of Tau aggregation. The Tau fragment reduced Tau-dependent cytotoxicity in a N2a cell model, suppressed the Tau-mediated neuronal dysfunction and ameliorated the defective locomotion in *C. elegans*. In vitro the fragment competes with full-length Tau for polyanionic aggregation inducers and thus inhibits Tau aggregation. Our combined in vitro and in vivo results suggest that the anti-aggregant Tau fragment may potentially be used to address the consequences of Tau aggregation in Tauopathies.

**Electronic supplementary material:**

The online version of this article (10.1007/s12035-018-1326-z) contains supplementary material, which is available to authorized users.

## Introduction

The aggregation of the microtubule-associated protein Tau is a hallmark of Alzheimer disease and a number of other neurodegenerative diseases collectively termed Tauopathies. The nature of the Tau species (monomers vs. oligomers vs. fibers) that are the real culprits remains a matter of debate, but the process of Tau aggregation is generally linked to neurodegeneration [1, 2]. Thus, suppressing Tau levels and Tau aggregation has emerged as a promising therapeutic approach for treatment of Tauopathies [3, 4].

Tau is a natively unfolded protein, which exhibits very little tendency for aggregation on its own. Despite the fact that Tau can be induced to aggregate in vitro with the aid of polyanions, the trigger for Tau aggregation in vivo still remains unclear. Nevertheless, it is known that the aggregation of Tau is regulated by two hexapeptide motifs with enhanced β-propensity (^275^VQIINK^280^ and ^306^VQIVYK^311^) in the second and third repeat of Tau [5]. Some Tau mutations in frontotemporal dementia (FTD) (e.g., ΔK280 & P301L) enhance β-propensity of the two hexapeptides and accordingly promote Tau aggregation[6]. By contrast, disruption of β-propensity via introducing two proline residues, known as β-structure breakers, into these hexapeptides (VQPINK & VQPVYK) prevents Tau aggregation and its toxicity [7]. Given their critical role in aggregation, the two hexapeptides become targets for developing inhibitors of Tau aggregation. Indeed, based on the structure of the two hexapeptides, a computer-aided design successfully identified several peptides showing high affinity to the two motifs, which inhibit Tau aggregation and thereby manifest therapeutic potential [8]. Similarly the inhibition of aggregation of other amyloidogenic proteins, such as Aβ, α-synuclein and prion protein can be achieved using β-sheet breaker peptides, i.e., peptides that are homologous to the targeted proteins but contain proline residues that interrupt the β-sheet structure [9, 10]. Although the introduction of proline into the hexapeptides can prevent self-aggregation of Tau in vitro and in vivo, it is not clear whether such β-breaker peptides of Tau can act as inhibitors of Tau aggregation and toxicity in cell and animal models.

Peptide-based therapy has been under consideration for some time; however, the nature of peptides—rapid degradation by proteases, low permeability across biological barriers (e.g., blood-brain barrier (BBB)) are intrinsic weaknesses that retard its therapeutic applications [11]. Nonetheless, recent years have seen a renaissance in gene therapy owing to the development of new technologies, e.g., genome editing tools (e.g., CRISPR-Cas9), and safer viral carriers (e.g., adeno-associated virus (AAV) and lentivirus) for delivery of genes into targeted cells (AAV) [12]. Our previous studies showed that the expression of anti-aggregant Tau in cultured cells or in transgenic mice does not cause overt side effects [13–15]. Thus in this study, we test whether a β-sheet breaker Tau fragment can inhibit Tau aggregation and thereby be potentially used for therapies for AD and other Tauopathies.

## Materials and Methods

### Cell Culture, Transfection and Treatments

The inducible Tet-On mouse neuroblastoma cell line (N2a) was generated as previously described [13]. The cells were cultured in Eagle’s minimum essential medium (MEM) supplemented with 10% fetal bovine serum (FBS), 0.1% nonessential amino acids, and 600 μg/ml G418. The expression of Tau was induced with 1 μg/ml doxycycline. Transfection of N2a cells were performed with lipofectamine 2000 (Invitrogen) according to the manufacturer’s manual. Twenty-four hours later, the conditioned medium was removed, and the cells were washed with warm PBS and then incubated in culture medium supplemented with 1 μg/ml doxycycline for 2 days to induce Tau expression.

### Protein Preparation

**Fig. 1:**
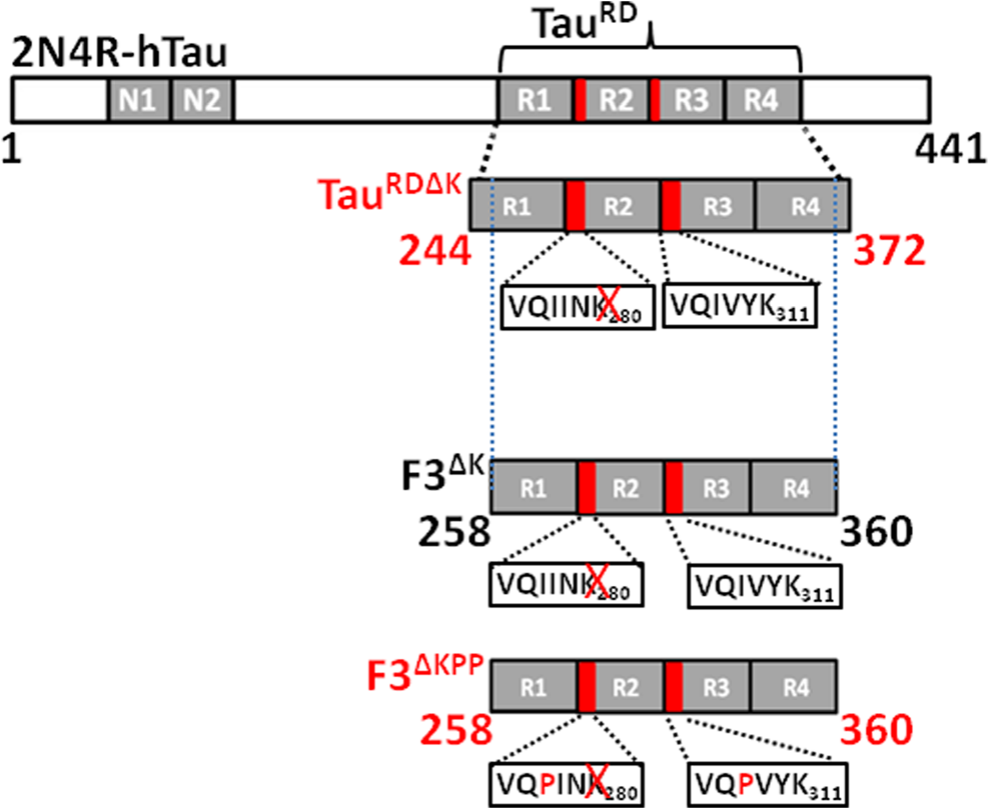
Constructs of Tau. The top bar diagram represents the longest isoform of the human Tau40 (441 residues). The diagram below hTau40 shows the four-repeat construct Tau^RD^. The two hexapeptides (_275_VQIINK_280_ and _306_VQIVYK_311_) are the motifs with the highest β-propensity at the beginning of the 2nd and 3rd repeat domains. The construct Tau^RDΔK^ contains the FTDP-17 mutation ΔK280 that accelerates aggregation by promoting the β-structure (pro-aggregant mutant). The construct F3^ΔK^ is a proteolytic Tau fragment composed of aa. 258–360 [17, 18]. The construct F3^ΔK-PP^ harbors ΔK280 and has two proline mutations (I277P and I308P in the hexapeptide motifs) that inhibit aggregation by disrupting the β-structure (anti-aggregant mutant)

Full-length Tau construct hTau40, Tau^RD^ construct (also known as K18, residues 244–372, comprising the four-repeat domain of Tau) harboring an FTDP-17 mutation ΔK280 (Tau^RDΔK^) and its fragment F3^ΔKPP^ (residues 258–360, harboring ΔK280 with I^177^ and I^308^ in the two hexapeptide motifs at the second and third repeat domain of Tau mutated to proline) were prepared as described previously [16, 17] (Fig. 1). Tau constructs were obtained in expression vector pNG2 (a derivative of pET-3a (Merck-Novagen), employing site-directed mutagenesis using the QuickChange site-directed mutagenesis method (Stratagene). Recombinant proteins were expressed in the *Escherichia coli* BL21 (DE3) strain (Merck-Novagen). The expressed proteins were purified from bacterial extracts by using the heat stability of Tau protein and by FPLC SP-Sepharose (GE Healthcare). The cell pellet was resuspended in extraction buffer (50 mM MES, 500 mM NaCl, 1 mM MgSO_4_, 1 mM EGTA, and 5 mM DTT, pH 6.8) supplemented with a protease inhibitor mixture (Roche Applied Science). The cells were disrupted with a French pressure cell and subsequently boiled for 20 min. The extracts were isolated by centrifugation, and the supernatant was dialyzed against cation exchange chromatography buffer A (20 mM MES, 50 mM NaCl, 1 mM MgSO_4_,1 mM EGTA, 2 mM DTT, and 0.1 mM PMSF, pH 6.8) for two times and loaded on a FPLC SP-Sepharose column. The protein was eluted with a linear gradient of cation exchange chromatography buffer B (20 mM MES, 1 M NaCl, 1 mM MgSO_4_, 1 mM EGTA, 2 mM DTT, and 0.1 mM PMSF, pH 6.8). The purity of proteins was ascertained by SDS-PAGE. Where necessary, breakdown products were removed by using the additional gel filtration column Superdex G75 with PBS buffer (137 mM NaCl, 3 mM KCl, 10 mM Na_2_HPO4, 2 mM KH_2_PO4, and 1 mM DTT, pH 7.4).

### ThS Fluorescence

Tau^RDΔK^ protein was dissolved at a concentration of 10 μM in PBS buffer supplemented with 2.5 μM heparin (Sigma, H3393, > 180 USP/mg, ~ MW 16 K), 1 mM dithiothreitol (DTT) and 40 μM thioflavine S (ThS). Different concentrations of F3ΔKPP (0, 10, 20, 40, and 80 μM) were mixed to the reaction mixture and the Kinetics of ThS fluorescence measured in a Tecan spectrofluorometer with an excitation wavelength of 440 nm and an emission wavelength of 521 nm (slit width, 2.5 nm each) in a black 384-well microtiter plate with round wells (Thermo Labsystems) using Magellan software. Measurements were carried out at 37 °C, and the background fluorescence was subtracted from respective blanks.

### Pelleting Assay

The aggregated samples were centrifuged at 61000 rpm (100,000×*g*; TLA.100.3 rotor) to generate pellet fraction of aggregated Tau protein. The pellet was resuspended in the same volume as supernatant. The samples were run on a 17% SDS-PAGE gel and the amount of Tau protein in the supernatants and pellets were quantified by densitometry of the Coomassie Brilliant Blue R-250 stained gels using ImageJ analysis software.

### Atomic Force Microscopy

One to two micromolar of Tau protein (after 24 h of Tau^RDΔK^ aggregation (10 μM)) was diluted in PBS and placed on freshly cleaved mica for 10 min. The excess unbound protein was washed with PBS three times and the mica was filled with imaging buffer (10 mM Tris-HCl, pH 7.4, 50 mM KCl). AFM imaging was performed in oscillation mode using a Nano Wizard Ultra-speed AFM microscope (JPK instruments) and Si3N4 cantilevers (NPS series, Bruker) with spring constants of 0.1–0.6 N/m. Drive frequency of the cantilever tip was set using in-built auto-tune option. Surface approach was performed at 0.7 V. Later on, to achieve minimal imaging forces between AFM stylus and sample and also to compensate for the thermal drift of the AFM, the amplitude set point was adjusted manually. The acquired images were processed using JPK data processing software.

### Electron Microscopy

Ten microliters of the samples (after performing the turbidity assay to monitor microtubule assembly) were incubated on glow discharged 200 mesh carbon-coated copper grids for 3 min followed by washing thrice with RB buffer and negatively stained with 2% filtered uranyl acetate for 30 s. Excess uranyl acetate was washed once with H_2_O. The specimens were examined with a JEOL electron microscope at 200 kV at the electron imaging facility of CAESAR. Images of the microtubules were captured with a CCD camera using EMMENU 4 software.

### Biochemical Assays

For solubility assays, cells were collected by centrifugation at 1000×*g* for 5 min. The levels and solubility of different Tau constructs were determined by sarkosyl extraction as previously described [17]. Supernatant and sarkosyl insoluble pellet samples were analyzed by Western blotting. The sarkosyl insoluble pellets and supernatants were loaded at 60:1 (pellet:supernatant). For quantification of Tau levels, the Western blots were probed with pan-Tau antibody K9JA (A-0024, DAKO, Glostrup, Denmark) and analyzed by densitometry.

### Cytotoxicity Assays

Cytotoxicity was assessed by a LIVE-DEAD assay kit (Molecular Probes, Eugene, OR). For the LIVE-DEAD assay, N2a cells seeded on the coverslips were induced to express Tau constructs for 2 days. EthD (5 mM; Molecular Probes) was added to the medium to a final concentration of 2 μM and incubated at 37 °C for 30 min. Cells were fixed with 4% paraformaldehyde in PBS for 15 min and processed for immunofluorescence.

### Immunofluorescence

Inducible N2a cells were either singly transfected with pBI5 plasmids encoding Tau^RDΔK^ or F3^ΔKPP^ or co-transfected with these two plasmids. After 1 day, cells were induced to express Tau with 1 μg/ml doxycycline for 2 days. The cells on the coverslips were fixed with 4% paraformaldehyde in PBS for 15 min, then permeabilized with 0.1% triton at room temperature for 10 min, incubated with 0.1% ThS for 5 min, and washed three times in 50% ethanol. Samples were blocked in 5% BSA for 1 h at room temperature, followed by incubation with the primary and secondary antibodies. Confocal images were captured with a LSM700 microscope (Zeiss, Oberkochen, Germany).

### Immunoprecipitation

Immunoprecipitation was done as described previously with slight modifications [17]. N2a cells were co-transfected with Tau^RDΔK^-His and F3^ΔKPP^ or hTau40 and F3^ΔKPP^ and induced to express Tau for 2 days. Transfected N2a cells were rinsed twice with ice-cold PBS, lysed in homogenization buffer (50 mM Tris-Cl, pH 7.4, 150 mM NaCl, 1% nonyl phenoxy polyethoxylethanol (NP-40), 10% glycerol, 1 mM ethylene glycol tetraacetic acid (EGTA), 20 mM NaF, 1 mM Na_3_VO_4_, 5 μM OA and protease inhibitor cocktail (Roche Applied Science,Basel, Switzerland) and incubated on ice for 30 min. After centrifugation at 16000×*g* at 4 °C for 20 min, the supernatant was collected and precleared with Dynabeads Protein G (Thermo Fisher Scientific; Dreieich, Germany) for 1 h at 4 °C. The lysates were incubated with control IgG or anti-His or DA9 antibodies overnight with constant rotation at 4 °C. Afterwards, Dynabeads Protein G was added to the lysates and incubated at 4 °C for 1 h. The beads were collected using a magnet and were washed four times with cold PBS, resuspended in Laemmli sample buffer, and analyzed by SDS-PAGE followed by Western blotting with Tau antibody K9JA.

For in vitro immunoprecipitation, recombinant proteins (50 μM hTau40, 200 μM F3^ΔKPP^ or a combination of both at 1:4 ratio) were incubated at 37 °C for at least 48 h in the presence or absence of 12.5 μM heparin (16 K). The reaction mixtures were incubated with control IgG or DA9 antibodies overnight with constant rotation at 4 °C in 1× Tris-buffered saline containing 0.05% Tween-20 Detergent (TBST). Afterwards, Dynabeads Protein G was added to the reaction mixtures and incubated at 4 °C for 3 h. The beads were collected using a magnet and were washed four times with cold 1× TBST, resuspended in Laemmli sample buffer, and analyzed by SDS-PAGE followed by western blotting with Tau antibody K9JA.

To detect the interaction between tau variants and heparin, recombinant proteins (50 μM hTau40, 200 μM F3^ΔKPP^ or a combination of both) were incubated at 37 °C for at least 48 h (or directly without incubation at 37 °C) in the presence or absence of 12.5 μM heparin (16 K). Afterwards, Dynabeads Protein G were added to the reaction mixtures directly without prior incubation with an antibody, and incubated at 4 °C for 3 h. The beads were collected using a magnet and washed four times with cold 1× TBST, resuspended in Laemmli sample buffer, and analyzed by SDS-PAGE followed by western blotting with Tau antibody K9JA.

### Turbidity Assays

Tau-induced microtubule assembly was monitored by 90° angle light scattering at 350 nm in a Fluorolog spectrophotometer (HORIBA). Ten micromolar PC-purified tubulin were mixed with 5 μM Tau protein in RB buffer (100 mM PIPES, pH 6.9, 1 mM DTT, 1 mM MgSO_4_, 1 mM EGTA, 1 mM GTP). Different concentrations of F3^ΔKPP^ (0, 5, 20, and 40 μM) were mixed to the reaction mixture and the polymerization started by transferring the ice-cold tubulin/Tau solution to the 37 °C warm cuvette holder at time point 0 min.

### *C. elegans* Methods

Pan-neuronal *snb-1* promoter (gift of Dr. B.C. Kraemer, Seattle, WA) was used to drive the expression of cDNA construct encoding the F3^ΔKPP^ fragment. Transgenic arrays expressing F3^ΔKPP^ fragment were generated by injecting *Psnb-1::F3*^*ΔKPP*^ (50 ng/μl) plasmid along with the selection marker *Pofm::dsRed* (50 ng/μl) (gift of Dr. Naoki Hisamoto, Nagoya University) into the gonad of N2 wild-type strain (Bristol). Integration of transgene arrays into *C. elegans* genome was achieved by UV irradiation (300 J/m^2^) and the resulting stable lines were out-crossed to N2 (Bristol) at least five times. Worm cultures were maintained according to the standard protocols [19]. Strains used were: PIR30: *pirIs30*[*Psnb-1::F3*^*ΔKPP*^*-low; Pofm::dsRed*], PIR31: *pirIs31*[*Psnb-1::F3*^*ΔKPP*^*-high; Pofm-2::dsRed*], CK10: *bkIs10*[*Paex-3::hTau1N4R* ^*V337M*^*; Pmyo-2::gfp*] (gift of Dr. B.C. Kraemer, Seattle, WA), PIR32: *pirIs30;bkIs10*, PIR33: *pirIs31;bkIs10*, CZ1197: *juIs73*[*Punc-25::gfp*]III (gift of Dr. E. Lundquist, Lawrence, KS), PIR34: *pirIs32;juIs73*, PIR35: *pirIs32;juIs73*, *jsIs609:Is:*[*Pmec-4::MLS::gfp*] (gift of Dr. Nonet, St Louis, MO), PIR36: *pirIs32;jsIs609,* PIR37: *pirIs33;jsIs609*, PIR5: *psnb-1:: pirIs5*[*Psnb-1::hTau40A152T-low;Pmyo-2::gfp*], PIR38: *pirIs5;pirIs31.*

### Behavioral Assay

The frequency of body bending (thrashes) was counted for 30 s after transferring the synchronized animals from each transgene in 20 μl of M9 buffer (22 mM KH_2_PO_4_, 42 mM Na_2_HPO_4_, 86 mM NaCl and 1 mM MgSO_4_) on a glass slide and allowing them to settle for 1 min [20]. For micrographs, 1-day old worms were allowed to crawl for 10 min and photographed using an Olympus SZH10 fitted with SC30 camera.

### Protein Extraction and Immunoblotting

For total worm lysates, 50 worms were dissolved in 30 μl 1× Laemmli buffer, boiled at 90 °C in a shaker for 10 min and loaded onto a 17% polyacrylamide gel for western blotting. To isolate the insoluble Tau, worm pellets were resuspended in high-salt RAB buffer [100 mM 2-(N-morpholino) ethanesulfonic acid (MES), 1 mM EGTA, 0.5 mM MgSO_4_, 20 mM NaF] and lysed by sonication (6 × 10 s, 10 s break) on ice. Lysates were centrifuged at 40000×*g* for 40 min to yield the resulting supernatant as the soluble RAB fraction. The RAB pellet was suspended in RAB + 1 M sucrose buffer, centrifuged for 20 min at 40000×*g*, and the supernatant was discarded. The resulting pellet, after brief washing, was solubilized in urea containing buffer (UREA) [30 mM Tris, 7 M urea, 2 M thiourea, 4% CHAPS (3-[(3-cholamidopropyl) dimethylammonio]-1-propanesulfonate), pH 8.5]. All buffers contained Complete Protease Inhibitor Mixture 3× (Sigma-Aldrich P8340, Hamburg, Germany), 1 μM Okadaic acid and 0.5 mM PMSF. Equal amounts of protein from each worm sample were loaded in separate gels, blotted and probed with antibodies against tau or the loading control tubulin. The following antibodies were used for immunoblotting: DM1α-tubulin (1:500; Sigma), K9JA (1:20,000; no. A0024; Dako), peroxide-conjugated secondary antibodies, and ECL solution (Thermo Scientific) were used to visualize the blots. AIDA software was used to perform densitometry.

### Survival Assay

To prevent mixing of the generations, worms were transferred every second day to freshly seeded NGM plates until the fertility period was over. Survival assay was carried out at 20 °C and worms were scored every 1–2 days until death with L4 stage annotated as day 0. Animals were judged as dead if they did not respond to a gentle touch or push.

### Imaging

Steady-state imaging of GFP-tagged mitochondria in mechanosensory neurons was performed by mounting worms on 2% agarose pads after anesthetizing in 50 mM sodium azide. Images were acquired at × 63 in two different regions, proximal part (~ 80 μm axonal part adjacent to cell body) and mid-region (beyond ~ 80 μm away from the cell body), using a Zeiss epifluorescence microscope equipped with a CCD (Photometrics) camera. To visualize the GABAergic motor neurons, young immobilized adults (15 mM sodium azide) were mounted on glass slides with 2% agarose pads and imaged at × 20 or × 40 using LSM 700 (Zeiss).

## Results

### **β-Sheet Breaker Tau Fragment (**F3^ΔKPP^**) Reduces Tau Aggregation**

**Fig. 2:**
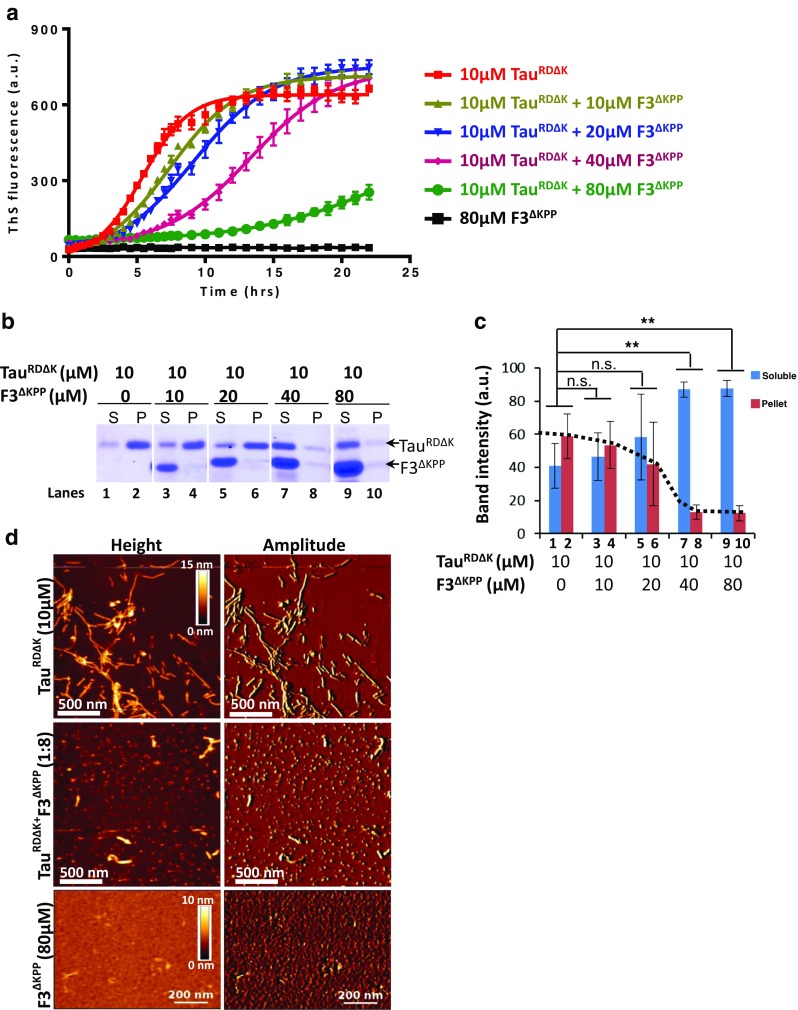
F3^ΔKPP^ reduces Tau^RDΔK^ aggregation in vitro. Tau^RDΔK^ (10 μM, upper band) was induced to aggregate with heparin (2.5 μM) in the absence or presence of different concentrations of F3^ΔKPP^ (lower band) for up to 24 h. **a** The extent of aggregation as measured by the thioflavin S fluorescence assay. All the measurements were performed in triplicate, *n* = 3. **b** Pellet assay showing the distribution of soluble and aggregated Tau^RDΔK^ (10 μM) alone (lanes 1, 2) or in the presence of F3^ΔKPP^ 10 μM (lanes 3, 4), 20 μM (lanes 5, 6), 40 μM (lanes 7, 8) and 80 μM (lanes 9, 10) at the end of incubation (S denotes the soluble fraction, P is the insoluble pellet fraction). **c** Densitometry quantification of the soluble (blue bars) and the insoluble (red bars) Tau^RDΔK^ from the gel shown in (b). Note the suppression of Tau^RDΔK^ aggregation at higher F3^ΔKPP^ concentrations (40 μM & 80 μM, red bars 8, 10). The results are from 3 different gels. One-way ANOVA was applied for multiple comparisons. Error bars denote SD. (ns, non-significant, **p < 0.001). **d** In vitro aggregation of Tau^RDΔK^ (10 μM) visualized using AFM in the absence (top panel) or presence (middle panel) of F3^ΔKPP^ (80 μM). 1–2 μM protein was diluted in PBS and placed on mica for imaging. No filamentous structures are seen in the presence of F3^ΔKPP^. F3^ΔKPP^ (80 μM) alone also does not form filamentous structures (bottom panel)

Our previous study had shown that the overexpression of the repeat domain of Tau harboring an FTDP-17 mutation ΔK280 (Tau^RDΔK^) in N2a cells results in the proteolytic generation of a fragment F3^ΔK^ (Fig. 1) [17]. We generated a β-sheet breaker Tau fragment via introduction of two prolines into the two hexapeptide motifs of F3^ΔK^, yielding F3^ΔKPP^ (Fig. 1). We tested whether recombinant F3^ΔKPP^ fragment can influence the aggregation of Tau^RDΔK^ in vitro using thioflavine S (ThS) to monitor Tau aggregation. F3^ΔKPP^ alone does not form aggregates at all (black curve, Fig. 2a), which is consistent with our previous studies showing that the introduction of two β-breaking prolines into the two hexapeptides disrupts Tau aggregation. The aggregation of Tau^RDΔK^ shows kinetics of nucleated assembly with an exponential phase followed by a plateau phase (red curve, Fig. 2a). F3^ΔKPP^ decreases the rate of Tau^RDΔK^ aggregation in a concentration-dependent manner. At low concentrations (10 μM and 20 μM), the effect is small, and roughly similar plateau values are reached (olive and blue curves, Fig. 2a). In contrast, higher concentrations (40 μM and 80 μM, purple and green) of F3^ΔKPP^ noticeably decrease the assembly rate.

To further confirm that F3^ΔKPP^ treatment affects the aggregation of Tau^RDΔK^, we separated soluble and insoluble Tau at the end of the incubation period via centrifugation and quantified their amounts by SDS-PAGE (Fig. 2b). When Tau^RDΔK^ was incubated alone, ~ 60% of the protein formed aggregates (Fig. 2b, lane 2 and Fig. 2c, bar 1, red). In agreement with the results of the Tau^RDΔK^ aggregation kinetic assay, addition of higher concentrations of F3^ΔKPP^ (40 μM and 80 μM) markedly reduces the aggregation of Tau^RDΔK^, such that only 10% of Tau^RDΔK^ appeared in the insoluble fraction (Fig. 2b, lanes 8 and 10 and Fig. 2c, bars 4,5, red). These results were corroborated by atomic force microscopy (AFM) analysis, which showed a reduction of fibrillar structures formed by Tau^RDΔK^ in the presence of F3^ΔKPP^ (Fig. 2d, compare top and middle panel). As expected, F3^ΔKPP^ alone did not show any fibrillary structures (Fig. 2d, bottom panel).

### F3^ΔKPP^ Reduces hTau40-Induced Microtubule Assembly

**Fig. 3:**
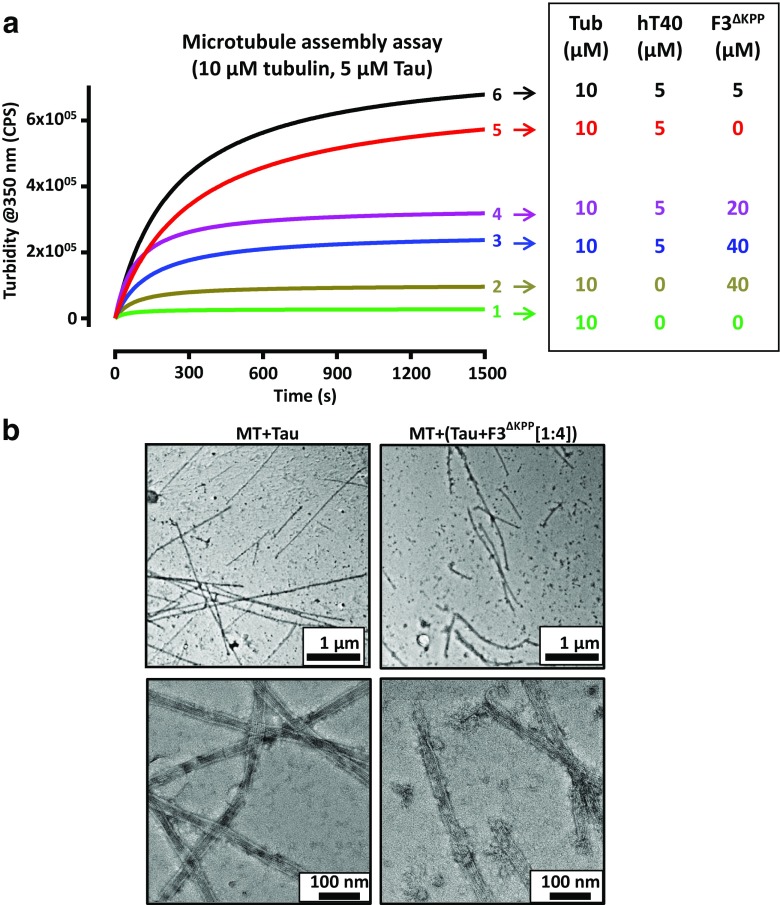
F3^ΔKPP^ slightly reduces hTau40-induced microtubule assembly. Microtubule assembly was measured by light scattering at 350 nm in the absence or presence of hTau40 with or without different concentrations of F3^ΔKPP^. **a** Tubulin and hTau40 concentration was 10 μM and 5 μM respectively. The ratios between the concentration of hTau40 and F3^ΔKPP^ were 1:1, 1:4, and 1:8. Note that F3^ΔKPP^ reduces hTau40-induced microtubule assembly by ~ 50% (curves 4 and 3, purple and blue). Tubulin without Tau (curve 1, *green*) or with F3^ΔKPP^ alone (20 μM; curve 2, ochre) does not assemble in these conditions either. **b** Microtubule (10 μM) assembly induced by Tau (5 μM) visualized by negative stain electron microscopy in the absence (left panel) or presence (right panel) of F3^ΔKPP^ (20 μM). Microtubules are reduced with F3^ΔKPP^ and become more fragile

Being a microtubule-associated protein, Tau plays an important role in microtubule assembly. We therefore examined whether F3^ΔKPP^ interferes with this physiological function of Tau. Microtubule polymerization assays were performed with or without different concentrations of F3^ΔKPP^. Tubulin (at 10 μM) without Tau served as a negative control, as it is unable to self-assemble into microtubules below the critical concentration (Fig. 3, curve 1, green). Similarly, F3^ΔKPP^ alone is also unable to induce microtubule assembly (Fig.3, curve 2, olive). In the presence of full-length Tau (5 μM hTau40), tubulin polymerizes within about 8 min (Fig. 3, curve 5, red). At 1:1 concentration (5 μM hTau40 + 5 μM F3^ΔKPP^), microtubule assembly was slightly more efficient (Fig. 3, curve 6, black). However, at higher concentrations of F3^ΔKPP^, the rate and extent of polymerization decreased in a concentration-dependent manner (Fig. 3, curve 4, purple for 20 μM F3^ΔKPP^ and curve 3, blue for 40 μM F3^ΔKPP^). This was verified by electron microscopy (EM), which showed less microtubules that were often shorter and decorated with protein clumps when Tau (5 μM) and F3^ΔKPP^ (20 μM) was added (Fig. 3b, right image) compared to Tau alone (Fig. 3b, left image).

### F3^ΔKPP^ Reduces Tau Aggregation and Cytotoxicity in a Cell Model of Tau Aggregation

**Fig. 4:**
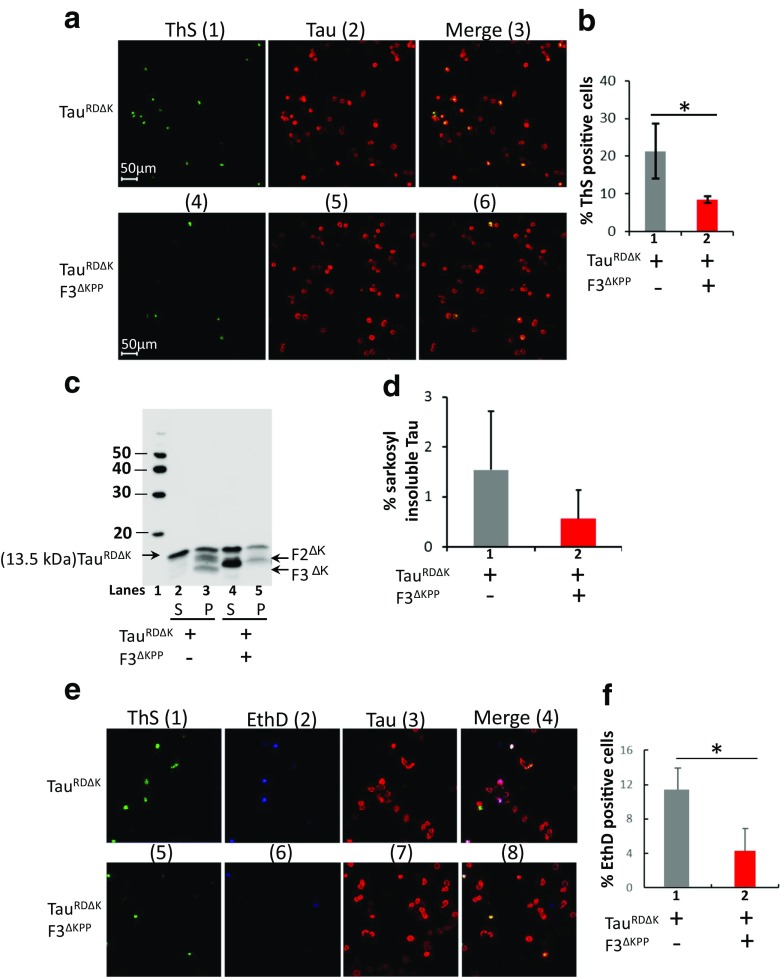
F3^ΔKPP^ reduces Tau^RDΔK^ aggregation and cytotoxicity in N2a cells. N2a cells were transfected with Tau^RDΔK^ or co-transfected with Tau^RDΔK^ and F3^ΔKPP^ for 2 days. **a** Thioflavin S (ThS) staining of Tau aggregates in N2a cells. Tau was monitored by immunostaining using a pan-Tau antibody K9JA (red panel 2 and 5). **b** Quantification of the ThS positive cells in relation to the Tau-expressing cells shown in (**a**). F3^ΔKPP^ strongly reduces ThS positive cells. (*t* test, *n =* 3; * *p* < 0.05). **c** Western blot analysis (17% PAGE, Tau antibody K9JA) of sarkosyl soluble (S) and insoluble (P) Tau^RDΔK^ in the absence (lanes 2, 3) or presence (lanes 4, 5) of F3^ΔKPP^. **d** Densitometry quantification of insoluble Tau^RDΔK^ (lanes 3 and 5) of the blot shown in (c). Note the strong reduction (~ 60%, red bar) of aggregated Tau^RDΔK^ by co-expression of F3^ΔKPP^. (unpaired *t* test, *n =* 6; *p* = 0.0624). **e** Cell death monitored by nuclear staining with Ethidium Homodimer (EthD). Tau expression was determined by immunolabeling with antibody K9JA (panel 3 and 7), Tau aggregation by ThS staining (green), and cell death by EthD staining (blue). Note: that cell death (blue) was dramatically reduced by the co-expression of F3^ΔKPP^ (*t* test, SD, *p < 0.05). **f** Quantification of cells positive for EthD staining shown in E. Cell death was reduced by the co-expression of F3^ΔKPP^ (*t* test, SD, *p < 0.05)

Next, we examined whether F3^ΔKPP^ can inhibit Tau aggregation in cells. It is known that Tau^RDΔK^ forms aggregates in N2a cells [17]. Therefore, we tested whether co-expression of F3^ΔKPP^ with Tau^RDΔK^ influences the aggregation of Tau^RDΔK^ in N2a cells. We used the ThS staining to visualize the Tau aggregates. When Tau^RDΔK^ is expressed alone, ~ 20% of cells are positive for ThS (Fig. 4a, upper panel 1–3, Fig. 4b, bar 1). The co-expression of F3^ΔKPP^ reduces the ThS positive cells to ~ 9% (Fig. 4a, bottom panels 4–6, Fig. 4b, bar 2). We also evaluated Tau aggregation using sarkosyl extraction to separate soluble and insoluble Tau. Consistent with our previous studies [18], the expression of Tau^RDΔK^ in cells results in its fragmentation (generating fragments F2^ΔK^, F3^ΔK^) which then nucleates Tau aggregation (Fig. 4c, lane 3). Notably, the co-expression of F3^ΔKPP^ inhibits the fragmentation of Tau^RDΔK^ by cellular proteases, as no F3^ΔK^ fragment was observed in the pellet (Fig. 4c, lane 5). Accordingly, the aggregation is reduced in the presence of F3^ΔKPP^ (Fig. 4d, bar 2 in red).

Our previous studies have shown that the aggregation of Tau induces cell death in N2a cells [17, 21]. We therefore assessed whether F3^ΔKPP^ could rescue Tau aggregation-induced cell death. We monitored cell death via nuclear staining with EthD. When Tau^RDΔK^ was expressed alone, 11% (11.1 ± 2.8%) of cells stained positive for EthD (Fig. 4e, upper panel 1–4, Fig. 4f, bar 1 in red). However, when F3^ΔKPP^ was co-expressed with Tau^RDΔK^, only 4.5% (4.5 ± 2.6%) of cells showed EthD staining (Fig. 4e, bottom panel 5–8, Fig. 4f, bar 2 in gray). Moreover, consistent with our previous studies, the majority of the ThS positive cells were stained by EthD (Fig. 4e 1,2,4 and Fig. 4e 5,6,8), pointing to cytotoxicity induced by Tau^RDΔK^ aggregation. Thus, F3^ΔKPP^ reduces Tau^RDΔK^-induced cytotoxicity via inhibiting its aggregation.

### F3^ΔKPP^ Suppresses Tau Pathology in an In Vivo *C. elegans* Model

**Fig. 5:**
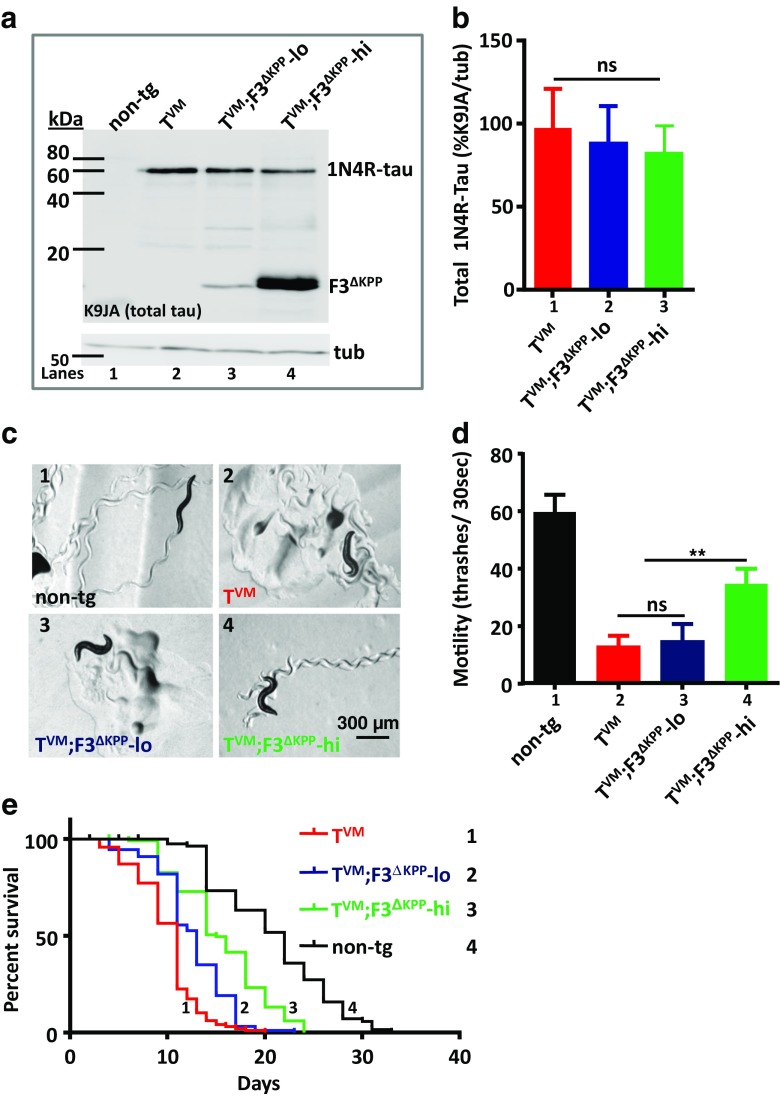
F3^ΔKPP^ at higher levels (F3^ΔKPP^-hi) improves the motor deficits in a *C. elegans* Tau aggregation model (T^VM^). F3^ΔKPP^ was expressed pan-neuronally in worms transgenic for human 1N4R-Tau^V337M^. T^VM^ expresses human 1N4R-Tau^V337M^ pan neuronally. T^VM^;F3^ΔKPP^-lo and T^VM^;F3^ΔKPP^ -hi are doubly transgenic for human 1N4R-Tau^V337M^ and F3^ΔKPP^ at low and high levels respectively. **a** Western blot of the total worm lysates from synchronized 1-day-old adults using pan-Tau antibody K9JA. Tubulin served as internal control. **b** Quantification of the total Tau levels. One-way ANOVA with Tukey’s test (*n* = 3, error bars denote SEM. ns, non-significant). **c** Micrographs showing tracks left behind by 1-day-old adults of the single and double transgenic worms. Non-transgenic (non-tg) served as control. **d** Body bending frequency (thrashes) of synchronized 1-day-old adults in liquid. Non-tg served as control, *n* = 40. One-way ANOVA with Tukey’s test was applied for multiple comparisons. Error bars denote SEM. (ns, non-significant, **p < 0.01). **e** Representative survival curves of single- T^VM^, double- T^VM^;F3^ΔKPP^-lo, and T^VM^;F3^ΔKPP^-hi transgenic worms, non-tg served as control. Mantel-Cox log-rank test was performed to determine the statistical differences between genotypes

Next, we turned to an in vivo model to test the protective efficacy of F3^ΔKPP^ fragment. We used the T^VM^ *C. elegans* model that expresses human 1N4R-Tau^V337M^ pan-neuronally [22]. This worm develops progressive motor dysfunction, neurodegeneration and accumulates detergent insoluble Tau aggregates. Since the protective effects of F3^ΔKPP^ were seen at higher stoichiometric ratios, we generated a transgenic *C. elegans* line F3^ΔKPP^-lo expressing F3^ΔKPP^ pan-neuronally at low levels, and another line F3^ΔKPP^-hi expressing F3^ΔKPP^ at higher levels. These lines were then individually crossed with T^VM^ resulting in double transgenic lines T^VM^;F3^ΔKPP^-lo and T^VM^;F3^ΔKPP^-hi. We first assessed total Tau levels in the parental T^VM^ worm and the double transgenic T^VM^;F3^ΔKPP^-lo and T^VM^;F3^ΔKPP^-hi worms, using the K9JA antibody which recognizes an epitope common to both the full-length Tau as well as the repeat fragment F3^ΔKPP^. Double transgenic T^VM^;F3^ΔKPP^-lo and T^VM^;F3^ΔKPP^-hi worms show comparable Tau levels as the single transgenic parental T^VM^ worm (Fig. 5a, lanes 2–4, Fig. 5b, bars 1–3).

T^VM^ worms show progressive motor dysfunction as seen by the distorted serpentine tracks left on the bacterial layer and lower thrashing rate when placed in liquid compared to non-transgenic control worms (compare Fig. 5c (1, 2) Fig. 5d, bars 1, 2). To check the protective effects of F3^ΔKPP^, we compared the motor function of these worms. F3^ΔKPP^ when co-expressed at higher levels in T^VM^;F3^ΔKPP^-hi results in improved motor function as seen by the near serpentine tracks left by this worm and higher thrashing rate in liquid compared to the parental worm T^VM^ (Fig. 5c4, Fig. 5d, bar 4). However, T^VM^;F3^ΔKPP^-lo worm that co-expresses F3^ΔKPP^ at lower levels, failed to show any improvement in the motility (Fig. 5c3, Fig. 5d bar 3). T^VM^ worms show reduced survival such that T^VM^ worms live ~ 50% shorter compared to non-transgenic worms (Fig. 5e curve 1, red; curve 4, black). In combination with F3^ΔKPP^-hi, however, 20% increase in the median survival of the T^VM^ worm is observed (Fig. 5e curve 3, green; Table 1).

**Table 1:** Statistical analysis of life-span assay performed as in Fig. 5e legend

Strain	Median survival	# deaths/total *N*	*p* value (vs non-tg)	*p* value (vs T^VM^)
Non-tg	22	70/120	–	< 10^−4^
T^VM^	11	100/120	< 10^−4^	–
T^VM^;F3^ΔKPP^-lo	12	98/120	< 10^−4^	< 10^−3^
T^VM^;F3^ΔKPP^-hi	15	103/120	< 10^−4^	< 10^−4^

**Fig. 6:**
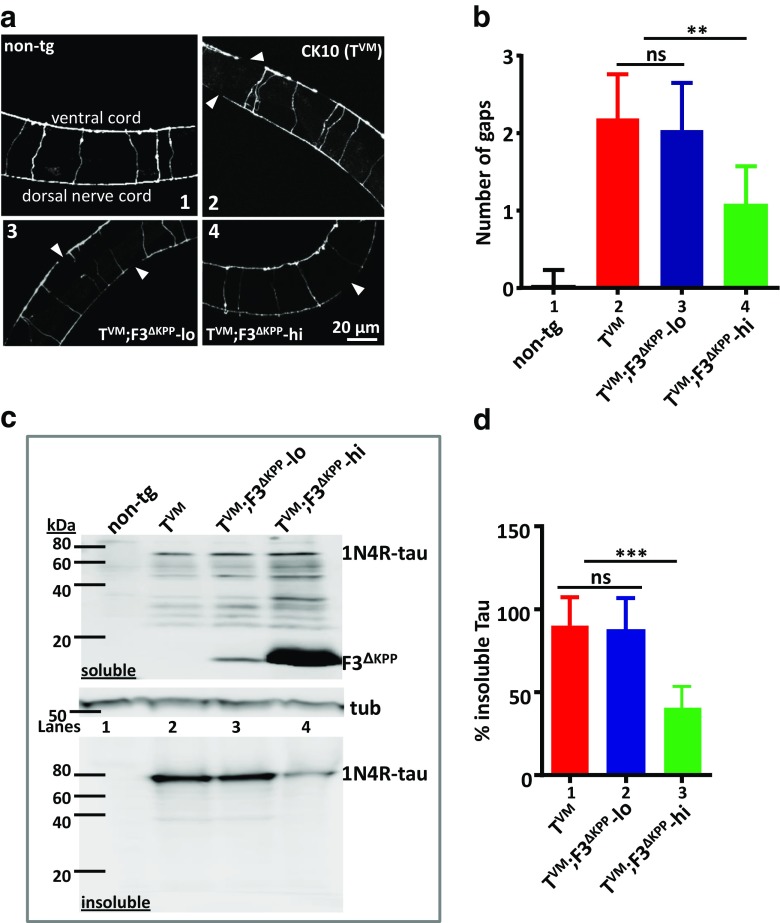
F3^ΔKPP^-hi reduces morphological defects and suppresses the Tau aggregation in T^VM^. **a** Fluorescence micrographs of GABAergic motor neurons in non-tg (1), T^VM^ (2), T^VM^;F3^ΔKPP^-lo (3), and T^VM^;F3^ΔKPP^-hi (4). Animals have ventral side oriented up. Arrowheads show gaps in the ventral and dorsal cord. **b** Number of gaps quantified in the neural cords of 1-day-old adults. Error bars denote SD. For comparison, one-way ANOVA with Tukey’s test was applied (*n* = 25, ns, not significant, **P < 0.01). **c** Sequentially extracted Tau from worm lysates of mixed stage animals resolved on 17% PAGE and immunoblotted using pan-Tau K9JA antibody. Tubulin served as a loading control. **d** Densitometry quantification of the insoluble Tau. T^VM^;F3^ΔKPP^-hi (insoluble panel, lane 4) shows reduced insoluble Tau (~ 50%). One-way ANOVA with Tukey’s test (*n* = 3, error bars denote SEM. ns, non-significant, ***P < 0.001)

GABAergic motor neurons that coordinate the motor functions in worms show a compromised integrity in the parental T^VM^ worm [22]. These neurons can be visualized by using a reporter transgene *juIs73*:[*Punc-25::gfp*]III [23] that expresses GFP specific to this subset of neurons (Fig. 6a). Thus, T^VM^, T^VM^;F3^ΔKPP^-lo, and T^VM^;F3^ΔKPP^-hi worms were crossed into this reporter strain to visualize the motor neurons in these worms. T^VM^ worms show significant neurodegeneration in the form of gaps in the dorsal and ventral nerve cords already at day 1 (~ 2 gaps) compared to non-tg reporter worms (~0.04 gaps). This does not differ from the T^VM^;F3^ΔKPP^-lo worms which also show a similar level of damage in these neurons (~ 1.96 gaps) (Fig. 6a (2, 3), Fig. 6b, bars 2,3). On the other hand, T^VM^;F3^ΔKPP^-hi worms with higher F3^ΔKPP^ levels show reduced neurodegeneration of the nerve cords (~ 1.08 gaps) (Fig. 6a4, Fig. 6b, bar 4). Thus an improvement in the integrity of motor neurons is consistent with an enhanced motility in T^VM^;F3^ΔKPP^-hi worms.

Since F3^ΔKPP^ inhibited Tau aggregation in vitro and in cell culture, we set out to investigate the status of insoluble Tau, a pathological hallmark of human Tauopathies that is also recapitulated by T^VM^ worms [22]. T^VM^ worms accumulate detergent insoluble Tau in their neurons. After extracting the soluble fraction, the insoluble Tau can be solubilized using a buffer with increasing solubilizing strength. Therefore, worm lysates from T^VM^, T^VM^;F3^ΔKPP^-lo, and T^VM^;F3^ΔKPP^-hi were sequentially extracted by homogenizing the respective worm pellets first in a high salt containing RAB buffer, resulting in the soluble Tau fraction. The remaining Tau fraction, which corresponds to the insoluble Tau, was then isolated using urea buffer (see “Materials and Methods”). T^VM^;F3^ΔKPP^-hi worms with higher F3^ΔKPP^ levels show a striking decrease in the detergent insoluble Tau aggregates compared to T^VM^ and T^VM^;F3^ΔKPP^-lo (Fig. 6c lower panel blot of insoluble protein, compare lane 4 with lanes 2, 3). Thus the fragment F3^ΔKPP^ shows protection by reducing the accumulation of insoluble Tau in T^VM^ worm neurons, in agreement with the results from in vitro and mammalian cell culture experiments described above.

**Fig. 7:**
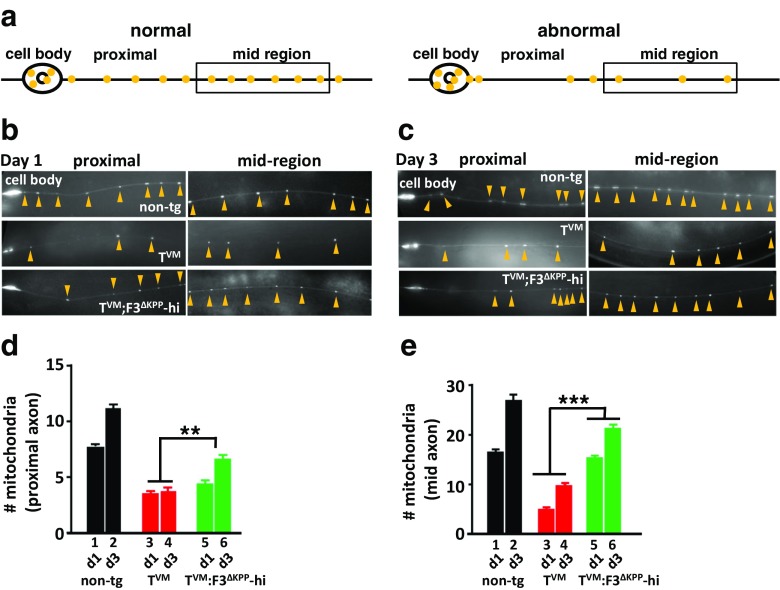
F3^ΔKPP^-hi improves the mitochondrial distribution in *C. elegans*. **a** Schematic representation of neurons with a normal and abnormal mitochondrial distribution. **b** Representative images of GFP tagged mitochondria in the mechanosensory neurons of non-tg reporter strain, T^VM^ and T^VM^;F3^ΔKPP^-hi animals at day 1 of adulthood. **c** Representative images of GFP-tagged mitochondria in the mechanosensory neurons of non-tg reporter strain, T^VM^ and T^VM^;F3^ΔKPP^-hi animals at day 3 of adulthood. **d** Average number of mitochondria quantified in the proximal axon (~ 80 μm axonal part adjacent to cell body) at days 1 and 3. Student’s *t*-test for comparison (error bars denote SEM. **P < 0.01). **e** Average number of mitochondria quantified in the mid-region of the axon (beyond ~ 80-μm length from cell body) at days 1 and 3. Student’s *t* test for comparison (error bars denote SEM. ***P < 0.001)

Tau (both soluble monomeric and insoluble aggregates) is known to interfere with the axonal traffic in cell culture and animal models [24, 25] which leads to a disrupted localization of axonal cargoes. The fact that the insoluble Tau levels are reduced in T^VM^;F3^ΔKPP^-hi worms prompted us to look at the mitochondrial distribution. We generated mitochondrial reporter strains using *jsIs609* worms [24] that express GFP-labeled mitochondria in six mechanosensory neurons. We performed the static imaging of GFP puncta in proximal and mid-region of the mechanosensory neurons in all three worm lines in 1- and 3-day-old adults, using a non-tg reporter strain as control (see “Materials and Methods”). Schematics in Fig. 7a depict neurons with a normal and an abnormal mitochondrial distribution. The mitochondrial distribution in the parental T^VM^ worm neurons differs from those in the non-transgenic reporter worms at both time points, with fewer mitochondria in proximal and mid regions of axons. Notably, the distribution is much more affected in the mid regions of axons towards the distal end (Fig. 7b, c compare top- and mid-panel, Fig. 7d, e, bars 1–4). Furthermore, F3^ΔKPP^ co-expression improves the mitochondrial distribution towards the wild-type level. Thus, T^VM^;F3^ΔKPP^-hi (Fig. 7b c compare mid- and lower panel, Fig. 7d, e, bars 3–6) but not T^VM^;F3^ΔKPP^-lo (Sup Fig. 1A, B compare mid- and lower-panel, Sup Fig. 1C, D 3–6) worms show an increased number of mitochondria in both the proximal and the mid-regions of neurons. We conclude that a reduced insoluble Tau load in combination with an improved cargo localization in the T^VM^;F3^ΔKPP^-hi neurons improves the motility of these worms.

### Peptide F3^ΔKPP^ Does Not Directly Interact with Other Tau Molecules in Cells

**Fig. 8:**
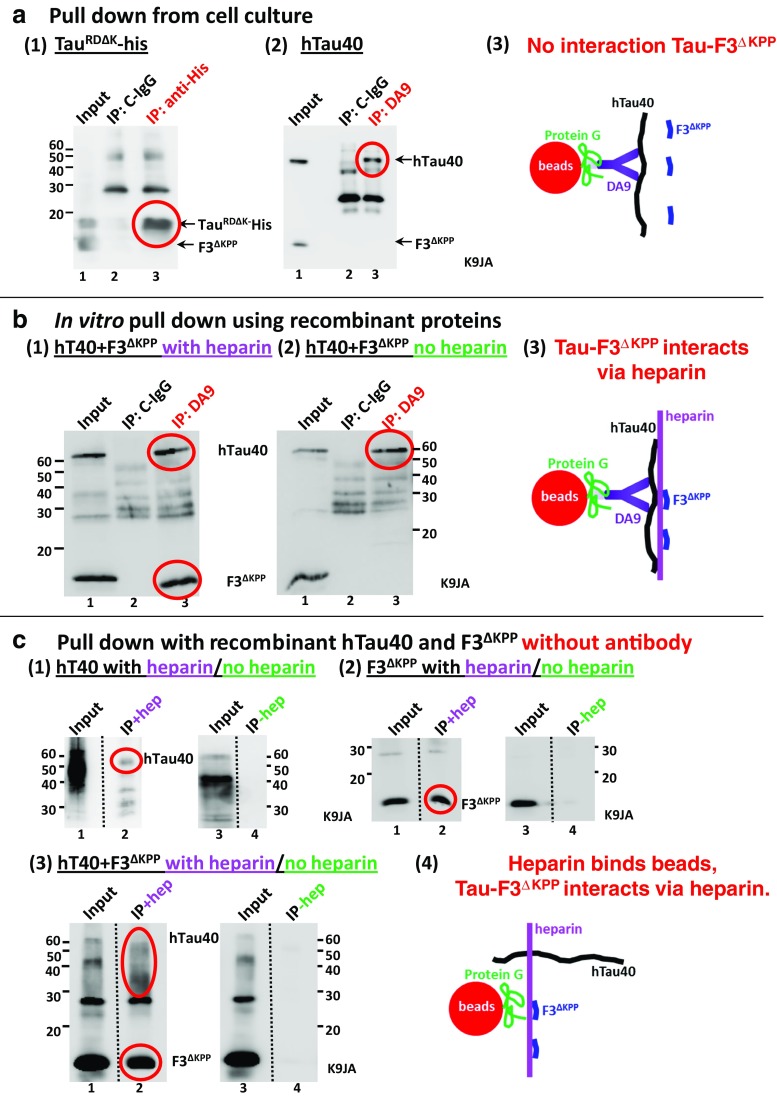
F3^ΔKPP^ does not interact directly with Tau40 or Tau^RDΔK^ to prevent aggregation. N2a cells were co-transfected with F3^ΔKPP^ and Tau^RDΔK^-His or hTau40 for 2 days. Antibody anti-His (blot A) and DA9 (epitope: aa. 112–129) (blot B) were used to immunoprecipitate Tau^RDΔK^-His or hTau40 in the cell lysates respectively, using non-specific IgG as control (A, B, lane 2). **a** Anti-His pulled down Tau^RDΔK^-His but not F3^ΔKPP^ (blot 1, lane 3). Similarly, DA9 pulled down hTau40 but not F3^ΔKPP^ (blot 2, lane 3), indicating that there is no direct interaction between F3^ΔKPP^ and hTau40. **b** Recombinant hTau40 (50 μM) and F3^ΔKPP^ (200 μM) were incubated at 37 °C for 48 h in the presence (blot 1) or absence (blot 2) of 12.5 μM heparin (M.W. 16 K) in BES buffer. Antibody DA9 (blots 1, 2, lane 3) was used to immunoprecipitate hTau40, using non-specific IgG as control (Ctrl-IgG, blots 1, 2, lane 2). Note that in the presence of heparin, DA9 pulls down both hTau40 and F3^ΔKPP^ (blot 1, lane 3). However, in the absence of heparin, DA9 pulls down only htau40 but not F3^ΔKPP^ (blot 2, lane 3), indicating an absence of a direct interaction between F3^ΔKPP^ and hTau40. **c** Heparin binds and pulls down F3^ΔKPP^ and hTau40 in the absence of antibody. Recombinant F3^ΔKPP^ and hTau40 at the same concentrations as described above were incubated at 37 °C for 48 h in the presence or absence of heparin (16 K) in BES buffer and Dynabeads Protein G added to the reaction mixtures afterwards. Note that hTau40 and F3^ΔKPP^ can be pulled down by heparin without requiring an antibody (blots 1, 2, 3, lane 2). In the absence of heparin, neither of the proteins is pulled down (blot 1, 2, 3, lane 4). Note the thick bands (red circle) corresponding to F3^ΔKPP^ in the pull-down lanes (blot 2, 3, lanes 2). This set of experiments shows (i) heparin is able to bind F3^ΔKPP^ and hTau40, (ii) heparin is able to bind the beads and thereby pull down both the proteins either individually or in combination, and (iii) the affinity is higher for F3^ΔKPP^ than hTau40. Hence, a direct interaction between F3^ΔKPP^ and hTau40 is absent, but the two interact indirectly via an aggregation inducer like heparin

Previously we showed that the pro-aggregant fragment F3^ΔK^ can nucleate and promote the aggregation of full-length Tau when co-expressed in N2a cells. Using coimmunoprecipitation, we further demonstrated that this occurs as a result of direct interaction between the two Tau species [17]. Since F3^ΔKPP^ can inhibit Tau^RDΔK^ aggregation, we asked whether this also results from a direct interaction with Tau^RDΔK^. We tested this through an immunoprecipitation assay. Since there is no antibody that can differentially recognize F3^ΔKPP^ and Tau^RDΔK^, we co-expressed F3^ΔKPP^ with Tau^RDΔK^-His in N2a cells, and pulled down Tau^RDΔK^-His with an antibody against the His tag. This pulled down Tau^RDΔK^-His, but not F3^ΔKPP^ (Fig. 8a, blot 1, lane 3, red circle), indicating that F3^ΔKPP^ does not interact with Tau^RDΔK^-His. Similarly, to confirm that F3^ΔKPP^ does not interact with other Tau variants, we co-expressed F3^ΔKPP^ with hTau40 in N2a cells and pulled down hTau40 with antibody DA9 that recognizes hTau40 but not F3^ΔKPP^. DA9 pulled down hTau40, but not F3^ΔKPP^ (Fig. 8a, blot 2, lane 3, red circle), suggesting that F3^ΔKPP^ does not interact with the full-length hTau40 either. These results show that the anti-aggregant F3^ΔKPP^ has a substantially different conformation than the pro-aggregant F3^ΔK^ which precludes a direct interaction.

To explain why F3^ΔKPP^ is able to inhibit aggregation without binding to hTau40 or Tau^RDΔK^ one has to consider the ternary system of polycationic molecules like hTau40, Tau^RDΔK^ or F3^ΔKPP^, and the polyanionic heparin. F3^ΔKPP^ competes with hTau40 or Tau^RDΔK^ for the aggregation inducer heparin and in the process sequesters it. To test this hypothesis, we performed in vitro immunoprecipitation assays using recombinant proteins. We incubated the recombinant 50 μM hTau40 and 200 μM F3^ΔKPP^ (ratio 1:4) at 37 °C in the presence or absence of heparin for 48 h, and pulled down hTau40 using the DA9 antibody. DA9 pulled down both proteins hTau40 and F3^ΔKPP^ only when the two were incubated in the presence of heparin, but not in the absence of heparin (Fig. 8b, blots 1, 2, lanes 3). These results suggest either that hTau40, F3^ΔKPP^ and heparin form a complex that can be pulled down by the antibody DA9 directed against hTau40, or that heparin binds to both Tau proteins and to the magnetic beads that leads to pull down of the F3^ΔKPP^-Tau core. To confirm this, the recombinant proteins—after incubating at 37 °C for 48 h with or without heparin—were pulled down, this time without an antibody. Surprisingly, both hTau40 and F3^ΔKPP^ were pulled down when heparin was present in the solution, alone (Fig. 8c, blots 1, 2, lanes 2, red circles) or in combination (Fig. 8c, blot 3, lane 2, red circles). In the absence of heparin, none of the proteins was pulled down, alone (Fig. 8c, blots 1, 2, lanes 4) or in combination (Fig. 8c, blot 3, lane 4). These results confirm that heparin binds the recombinant proteins hTau40 and F3^ΔKPP^, and is able to pull down both of them by binding to the magnetic beads. Notably, heparin pulls down F3^ΔKPP^ almost completely compared to hTau40 as seen by the thick bands at F3^ΔKPP^ position in the pull-down lanes (compare in Fig. 8c lane 2 in blots 1, 2, red circles).

Furthermore, direct pull-down experiments without prior incubation of reaction mixtures at 37 °C led to the pull-down of F3^ΔKPP^ by heparin and not full-length Tau (Sup Fig. S2, red circle). These results show that F3^ΔKPP^ binds heparin with a higher affinity than full-length Tau, consistent with its higher specific positive charge. We therefore conclude that F3^ΔKPP^ inhibits the aggregation of Tau^RDΔK^ or hTau40 by a competition and sequestration effect whereby F3^ΔKPP^ preferentially binds and engages the aggregation inducers like heparin. The effective reduction of aggregation inducers then results in the inhibition of Tau^RDΔK^ or hTau40 aggregation.

## Discussion

Tau aggregation characterizes Tauopathies including AD [26]. Numerous publications have reported that Tau aggregation causes or accompanies a neurotoxic process, though the precise nature of this remains a matter of debate [2]. Accordingly, suppressing Tau aggregation has long been proposed to be a therapeutic approach for AD and other Tauopathies. The development of low molecular weight inhibitors of Tau aggregation has been challenging because of Tau’s variable conformations and because protein-protein interaction interfaces are generally flat and large, contrary to the deep cavities that small molecules can bind to [27]. During the past decade, several types of low MW compounds have been shown to inhibit Tau aggregation in vitro and in vivo [24, 28–31]. Methylene blue and its derivatives even entered clinical trials, although finally failed at phase III [32]. The caveat is that compounds may stabilize rather than disrupt the low-n oligomers (likely the more toxic species), if they form binding pockets for low MW compounds [33]. The increasing evidence that Tau oligomers are the most toxic species [34–36] may explain why low MW Tau aggregation inhibitors have not yet succeeded in clinical trials. There is thus a need to search for alternative Tau aggregation inhibitors. Indeed, several groups are developing peptides targeting the two hexapeptide motifs of Tau that govern the Tau aggregation process [8, 37, 38]. The advantage to this approach is that the peptides occupy larger and more specific interaction interfaces between Tau molecules than low MW compounds, necessary to inhibit the overall aggregation process.

Here, we show that a β-structure breaker Tau fragment (F3^ΔKPP^) inhibits Tau aggregation and reduces Tau-induced cytotoxicity in vitro and in vivo (Figs. 2, 4, 5, 6 and 7). Surprisingly, F3^ΔKPP^ does not inhibit Tau aggregation via binding to Tau molecules, as no direct interaction of F3^ΔKPP^ with other Tau molecules was observed in vitro or in cultured cells (Fig. 8). Therefore, it is likely that the inhibition of aggregation is due to competition between F3^ΔKPP^ and Tau for aggregation inducers instead of a direct interaction of F3^ΔKPP^ with Tau molecules. Tau contains multiple heparin binding sites as deducted from experiments using truncated Tau constructs, but the repeat region is indispensable for aggregation induced by heparin [39]. Despite being a derivative of the Tau repeat domain, F3^ΔKPP^ is incapable of aggregation due to proline substitutions in the two hexapeptide motifs that act as β-sheet breakers [5, 7]. Nevertheless, F3^ΔKPP^ is expected to have a similar affinity for polyanions like heparin. Indeed, in this study we show that F3^ΔKPP^ physically interacts with heparin. Notably, the F3^ΔKPP^ and heparin interaction is stronger than the full-length Tau and heparin interaction (Fig. 8c, blot 2, lane 2, compare with blot 1, lane 2, red circles). Thus, in a scenario where aggregation is induced by polyanions like heparin, F3^ΔKPP^ preferentially binds and sequesters such aggregation inducers and thereby, the aggregation process is halted.

The factors causing Tau aggregation in vivo remain a matter of debate. Post-translational modifications have been reported to contribute to Tau aggregation. However, it is unclear if they alone are sufficient to initiate Tau aggregation, given that no in vitro studies show the formation of abundant Tau fibrils by post-translationally modified Tau in the absence of cofactors [40]. Similarly, RNA molecules are capable of inducing Tau aggregation [41]. Recent studies show that Tau forms part of the stress granule pathway, which under pathological conditions leads to irreversible aggregation of RNA binding proteins [42, 43]. The interaction of Tau with ribosomes can inhibit protein synthesis and this interaction is based on RNA’s and RNA binding proteins [44]. Besides, Tau may undergo reversible phase transition in cells in the presence of various cofactors [45, 46]. This phase transition from free soluble tau to liquid droplets might indeed represent early phases in the tau aggregation process. Other factors that could potentially induce Tau aggregation in vivo include sulphated glycosaminoglycans like heparin sulphate, chondroitin sulphate and dermatan sulphate. In the Alzheimer Disease brain, heparin sulphate coexists with Tau in tangle bearing neurons [47, 48] and neurons lacking neurofibrillary tangles may stain positive for hyperphosphorylated Tau [39]. Moreover, reports that sulphated glycosaminoglycans promote Tau phosphorylation by several kinases [49–51] and also prevent Tau binding to microtubules [52], suggest multiple effects favoring Tau aggregation.

**Fig. 9:**
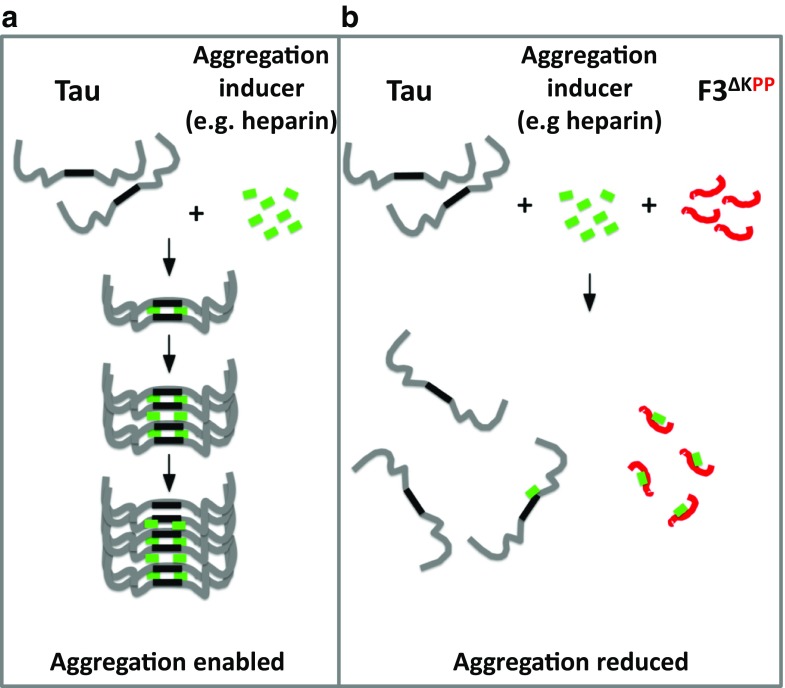
Model of Tau aggregation and competition with anti-aggregant F3^ΔKPP^. Tau aggregation can be induced in vitro or in cells by cofactors such as heparin, RNA, or other polyanions (a). In such a scenario, F3^ΔKPP^ can compete with Tau molecules by preferentially binding and sequestering the aggregation inducers. This might prevent the formation of early oligomers (dimers, trimers etc.) (b). At low F3^ΔKPP^ levels, sufficient inducers are available so that the aggregation of Tau may not be disturbed. With increasing F3^ΔKPP^ levels, the inducers available for Tau are reduced and the aggregation of Tau is retarded

Under physiological conditions, Tau prefers to bind to MT because of specific interactions, in addition to the electrostatic interactions of oppositely charged polymers [53]. Furthermore, this interaction of Tau with MT actually prevents interaction between the β-structure forming parts of Tau and thereby prevents self-assembly of Tau [54]. Under pathological conditions, the affinity of Tau to MT can be reduced by post-translational modifications (e.g., phosphorylation). As such, Tau may be induced to aggregate by inducers like RNA and/or heparin sulphate. Hence F3^ΔKPP^, unable to contribute to filamentous assembly due to absence of β-structure, can instead compete with other Tau molecules for these aggregation inducers. Thus, when the concentration of F3^ΔKPP^ is low, the amount of the available inducers (e.g., heparin, RNA, or other cellular polyanions) may be sufficient to trigger the aggregation of other Tau molecules, but with increasing F3^ΔKPP^ levels the inducers available for other Tau molecules are reduced, resulting in the reduction of aggregated Tau (Fig. 9). In conclusion, we revealed that a β-structure breaker Tau fragment (F3^ΔKPP^) can inhibit Tau aggregation and Tau-induced cytotoxicity. This β-structure breaker Tau fragment may have potential as a therapeutic approach for Tauopathies.

## Electronic supplementary material

ESM 1 Fig. S1: F3ΔKPP-lo has no effect on the mitochondrial distribution. (A) Representative images of GFP tagged mitochondria in the mechanosensory neurons of TVM and TVM;F3ΔKPP-lo animals at Day 1 of adulthood, non-tg reporter strain serves as control. (B) Representative images of GFP tagged mitochondria in the mechanosensory neurons of TVM and TVM;F3ΔKPP-lo animals at Day 3 of adulthood, non-tg reporter strain serves as control. (C) Average number of mitochondria quantified in the proximal axon (~80 μm axonal part adjacent to the cell body) at day 1 and 3. Student t-test for comparison (error bars denote SEM. *p < 0.05). (D) Average number of mitochondria quantified in the mid-region of the axon (beyond ~80 μm length from the cell body) at day 1 and 3. Student t-test for comparison (error bars denote SEM. ns, not significant). Fig. S2: Heparin has a higher affinity to F3ΔKPP than Tau. Pull-down experiments of recombinant proteins (hTau40 and/or F3ΔKPP) directly without prior incubation of the reaction mixtures at 37 °C in the presence or absence of heparin. (A) Heparin does not pull down hTau40 in a direct pull-down experiment (blot 1, lane 2). No hTau40 is pulled down without heparin in a direct pull-down experiment as expected (blot 2, lane 2). (B) Heparin pulls down F3ΔKPP but not htau40 in a direct pull-down experiment (blot 1, lane 2; red circle), suggesting a preferential binding of heparin to F3ΔKPP. Neither hTau40 nor F3ΔKPP is pulled down without heparin in a direct pull-down experiment as expected (blot 2, lane 2). (PDF 2.34 mb)

## Abbreviations

2N4R-Tau: isoform 2, largest isoform of human Tau in CNS (441 residues, 2 inserts +4 repeats), accession: NP_005901.2, amino acid numbering based on full-length human 2N4R isoform
1N4R-Tau: human Tau isoform 5 in CNS (412 residues, 1 insert +4 repeats), accession: NP_001116539.1, amino acid numbering based on full-length human 2N4R isoform
AFM: atomic force microscopy
ANOVA: analysis of variance
EM: electron microscopy
F3^ΔKPP^: Tau repeat domain fragment with ∆K mutation and two proline substitutions in the hexapeptide motifs (258–360 residues)
MAPT: microtubule-associated protein Tau
non-Tg: non-transgenic
T^VM^: worms expressing mutant V337 M human 1N4R-Tau
T^VM^;F3^ΔKPP^-hi: double transgenic worm expressing T^V337M^, and F3^ΔKPP^ at higher levels
T^VM^;F3^ΔKPP^-lo: double transgenic worm expressing T^V337M^, and F3^ΔKPP^ at lower levels
Tau^RDΔK^: Tau repeat domain with ∆K280 mutation (244–372 residues, 4 repeat)
ThS: thioflavine S
VNC: ventral nerve cord
