# Supplementary material for: Suppressing Tau Aggregation and Toxicity by an Anti-Aggregant Tau Fragment
Source: Mol Neurobiol. 2018 Sep 8;56(5):3751–67. doi: 10.1007/s12035-018-1326-z (PMC6476873; doi:10.1007/s12035-018-1326-z)
Supplement: Supplementary file 1 — Fig. S1: F3ΔKPP-lo has no effect on the mitochondrial distribution. (A) Representative images of GFP tagged mitochondria in the mechanosensory neurons of TVM and TVM;F3ΔKPP-lo animals at Day 1 of adulthood, non-tg reporter strain serves as control. (B) Representative images of GFP tagged mitochondria in the mechanosensory neurons of TVM and TVM;F3ΔKPP-lo animals at Day 3 of adulthood, non-tg reporter strain serves as control. (C) Average number of mitochondria quantified in the proximal axon (~80 μm axonal part adjacent to the cell body) at day 1 and 3. Student t-test for comparison (error bars denote SEM. *p < 0.05). (D) Average number of mitochondria quantified in the mid-region of the axon (beyond ~80 μm length from the cell body) at day 1 and 3. Student t-test for comparison (error bars denote SEM. ns, not significant). Fig. S2: Heparin has a higher affinity to F3ΔKPP than Tau. Pull-down experiments of recombinant proteins (hTau40 and/or F3ΔKPP) directly without prior incubation of the reaction mixtures at 37 °C in the presence or absence of heparin. (A) Heparin does not pull down hTau40 in a direct pull-down experiment (blot 1, lane 2). No hTau40 is pulled down without heparin in a direct pull-down experiment as expected (blot 2, lane 2). (B) Heparin pulls down F3ΔKPP but not htau40 in a direct pull-down experiment (blot 1, lane 2; red circle), suggesting a preferential binding of heparin to F3ΔKPP. Neither hTau40 nor F3ΔKPP is pulled down without heparin in a direct pull-down experiment as expected (blot 2, lane 2). (PDF 2.34 mb) [file 12035_2018_1326_MOESM1_ESM.pdf]

Supplemtal Fig. S1

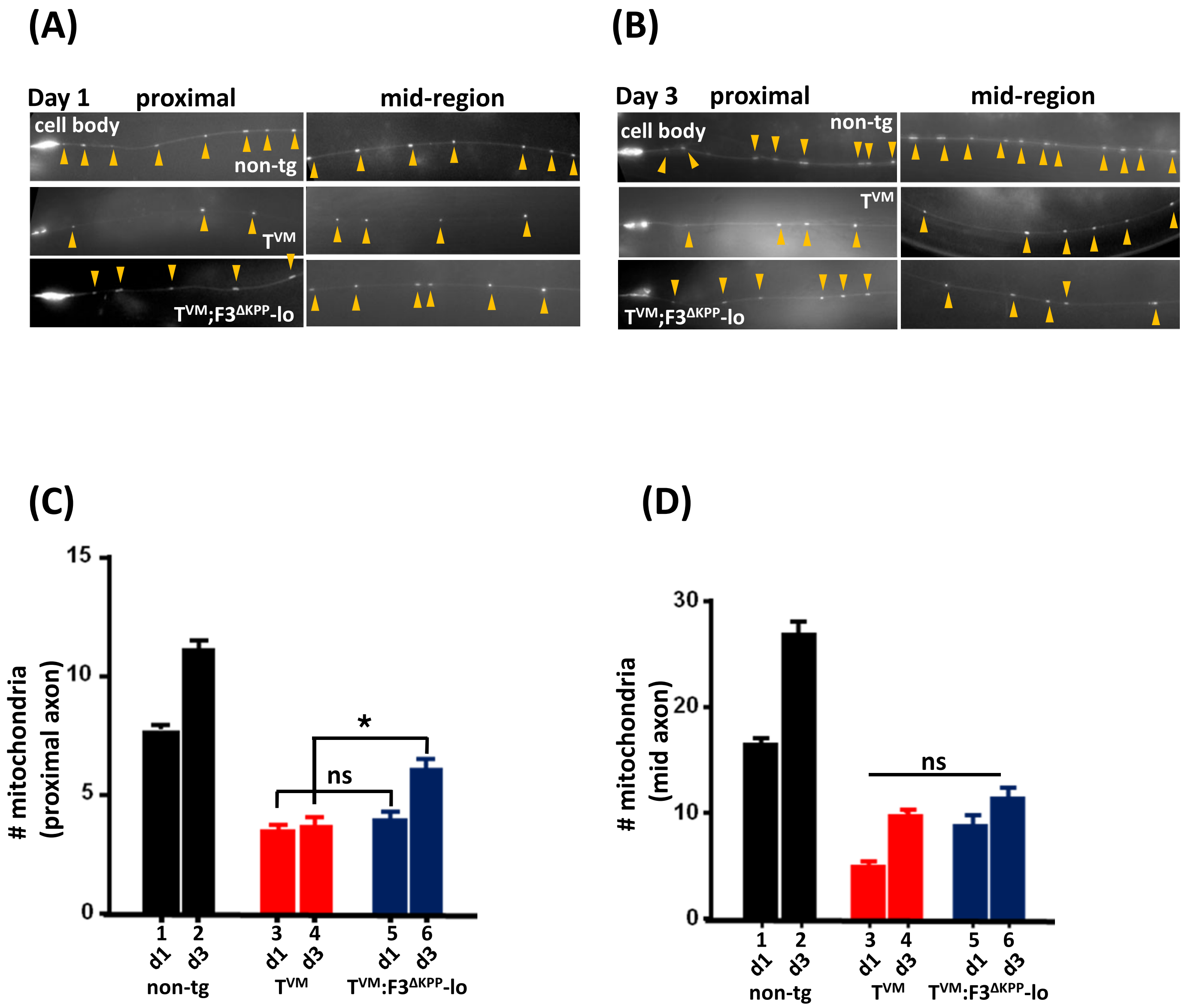

F3$^{\Delta KPP}$-lo shows no effects on Tau-induced defects in mitochondrial transport *in vivo*.

Supplemental Fig. S2

Direct pulldown with heparin without prior incubation at 37˚C

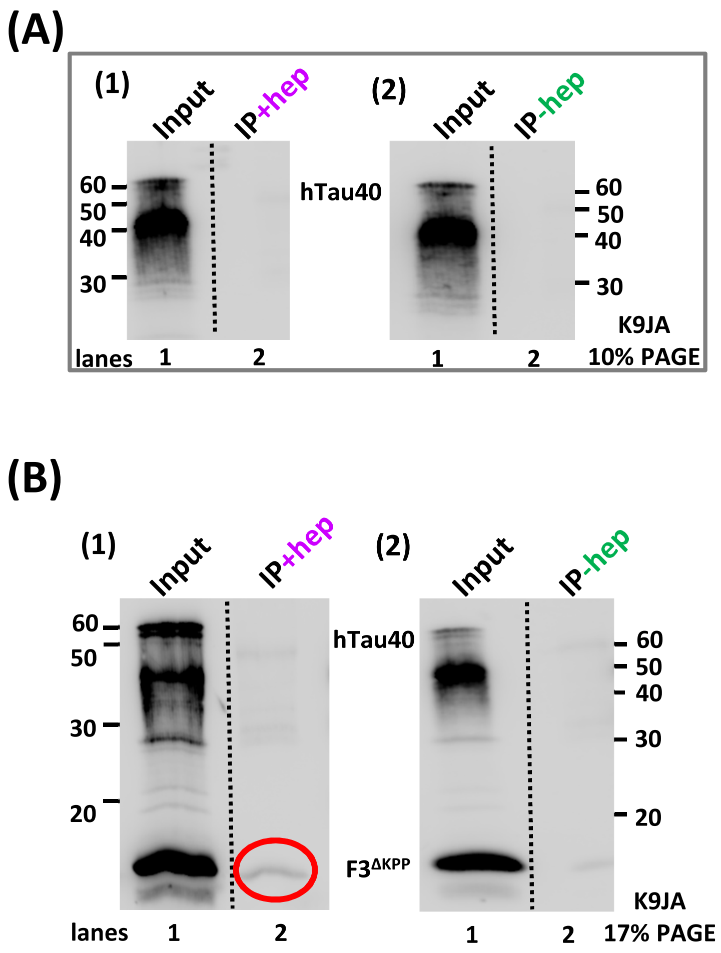

$F3^{\Delta KPP}$ not full-length Tau binds heparin in a pulldown without a prior incubation at 37˚C in vitro.
